# Structural and Convergent Properties of Structured Assessment of Protective Factors (SAPROF) Ratings in a Treated Sexual Offending Sample

**DOI:** 10.1177/10731911251381890

**Published:** 2025-11-12

**Authors:** Leezan Alawes, Mark E. Olver

**Affiliations:** 1 University of Saskatchewan, Saskatoon, Canada

**Keywords:** factorial validity, convergent validity, confirmatory factor analysis, exploratory structural equation modeling, Structured Assessment of Protective Factors

## Abstract

The Structured Assessment of Protective Factors (SAPROF) is a measure of protective factors intended to augment violence risk assessment. While prior research supports the predictive validity of SAPROF ratings, factorial and convergent validity have been underexamined, each of which is required to ensure that the instrument measures intended targeted constructs and converges with test scores from established measures. We evaluated the structural and convergent properties of SAPROF ratings through examining its factor structure and convergence with measures of relevant constructs, as a function of ethnocultural heritage, in a treated sample of 461 men with sexual offense conviction histories. The SAPROF was rated from institutional files pre-and posttreatment. Results of exploratory structural equation modeling (ESEM) of pre and post SAPROF item ratings identified a temporally stable three-factor model that was invariant across ethnocultural groups; however, it departed from the developers’ original subscale structure—the factors were termed Internal-Prosocial, Motivational-Lifestyle, and External—to reflect continuity with, yet departure from, the current subscale structure. SAPROF ratings were correlated in theoretically and clinically meaningful ways with scores on relevant risk-need-responsivity (RNR) measures. The results support the structural and convergent validity of SAPROF ratings and identified a slightly modified subscale structure in the present sample.

Historically, the formalized assessment of static and dynamic risk factors has taken precedence in risk assessment, intervention, and management in forensic and correctional settings. And for good reason; risk factors are fundamental in aiding the evaluation of an individual’s potential to (re)engage in new acts of crime and violence, they can guide where to intervene in service delivery, and inform efforts at risk management to prevent recidivism. Increasingly, however, clinicians and researchers have been proposing that the sole and exclusive focus on risk factors is implicitly negatively biased in forensic psychological practice, given the emphasis on deficits and problem areas, to the relative neglect of strengths and resiliencies (de Ruiter & Nicholls, 2011; de Vries Robbé et al., 2011; Rennie & Dolan, 2010). Protective factors, by contrast, serve a positive countering effect to risk factors and their characteristics are associated with a lower likelihood of negative outcomes (i.e., recidivism) promoting resilience, rehabilitation, and positive outcomes in individuals involved in the legal system (de Vries Robbé et al., 2011). That is, the introduction of protective factors in risk assessments can counter the potential for bias in assessments by creating a shift toward a strength-based approach as they can be clinically valuable for promoting the reduction of recidivism, strengthening the working alliance across services, and providing goals for treatment to motivate change (de Vries Robbé, de Vogel, et al., 2015). Protective factors have the potential to mitigate risk of a violent outcome while simultaneously assisting in the understanding of desistance (i.e., the cessation of criminal offending; de Vries Robbé et al., 2013, 2016; Serin et al., 2016).

It bears some emphasis that the presence of protective factors does not amount to the absence of risk factors; rather, in relation to risk variables, scholars have postulated that protective factors are considered to exist on two sides of the same coin (de Vogel et al., 2011; de Vries Robbé, de Vogel, et al., 2015). For instance, indicators of employment and education can have protective effects as a history of employment and average or better cognitive-academic ability tend to be linked to a decrease in violent outcomes, while their alternatives (i.e., unstable employment and low educational attainment) have been considered risk factors for treatment attrition and subsequent recidivism (Coupland & Olver, 2020; Kennedy-Turner et al., 2020; Nikulina & Widom, 2019; Olver et al., 2011; Porter et al., 2023; Ttofi et al., 2016). This implies that protective and risk factors may exist on a continuum and, at least in principle, can exist simultaneously, or per de Vries Robbé, de Vogel, et al.’s (2015) argument, represent opposite sides of the same coin.

## The Structured Assessment of Protective Factors (SAPROF)

The Structured Assessment of Protective Factors (SAPROF; de Vogel et al., 2009, 2011) is a formalized measure of protective factors that systematically and comprehensively evaluates a range of strengths and resources that can mitigate the risk of reoffending and promote positive outcomes (e.g., coping, work, social network). The SAPROF was developed to be used in conjunction with an established structured forensic risk assessment tool as a means to comprehensively assess risk for violent or sexual offending in adult correctional and forensic populations. Its use is intended to guide recidivism prevention efforts by informing treatment planning and community reintegration (de Vogel et al., 2011; de Vries Robbé et al., 2016) across adult correctional and forensic populations. The items are arranged into three subscales, Internal (personal characteristics that can be protective such as positive coping), Motivational (factors indicating a motivation to prosocially participate in society such as positive attitudes toward authority), and External (protective factors external to the individual, such as living circumstances; de Vogel et al., 2011).

More than a decade of validation research has demonstrated SAPROF ratings to have good predictive accuracy for nonrecidivism. The inaugural SAPROF predictive validity study (de Vries Robbé et al., 2011) in a sample of 105 adult male Dutch forensic psychiatric patients with an average treatment length of 5.3 years found SAPROF total scores had high predictive validity (accuracy for sexual recidivism [AUC] = .80) for 1, 2, and 3-year violent nonrecidivism after discharge. Further, de Vries Robbé et al. (2011) found integrating SAPROF ratings with scores from a general violence risk measures (Historical Clinical Risk-20, HCR-20; Douglas et al., 2011) improved prediction beyond HCR-20 ratings alone. Subsequent replications of the SAPROF have provided further support for its predictive properties across varied international samples and settings. For instance, de Vries Robbé, de Vogel, et al. (2015), in a sample of 83 male sexual offending persons, found SAPROF ratings predicted decreased sexual recidivism. In Austria, Yoon et al. (2018), in a sample of 450 Austrian sexual offending persons rated on the SAPROF and a dynamic sexual violence risk tool (Sexual Violence Risk-20, SVR-20; Boer et al., 1997) also found SAPROF ratings to be associated with decreased recidivism, and for the tandem of tools to augment predictive validity. Elsewhere, in a Canadian study, Coupland and Olver (2020) found that SAPROF scores were inversely associated with community recidivism and that risk and protective factor scores predicted positive community outcomes. Kashiwagi et al. (2018), in a forensic sample in Japan, found SAPROF and HCR-20 combined ratings provided a better indicator for predictive validity for desistance from violent (and sexual) offending than HCR-20 scores alone, akin to de Vries Robbé et al. (2011). Most recently, a meta-analysis of the SAPROF’s predicative validity for crime and violence demonstrated medium effects (*d* = .51–.77) across 39 validation studies (Burghart et al., 2023). Treatment-related changes in SAPROF ratings were also associated with decreased violent and general recidivism (Burghart et al., 2023).

Much less research has examined the structural properties of SAPROF ratings, and specifically, the veracity of its three-subscale structure. In an Australian sample of 201 treated men with violence conviction histories, Klepfisz et al. (2020) did a conjoint analysis of selected SAPROF and HCR-20 item ratings through structural equation modeling, finding that conceptually similar risk and protective items loaded onto distinct, but correlated factors. There was weak evidence, however, for the factorial validity of SAPROF item ratings for the measure’s traditional three subscale structure.

## Situating Protective Factors Within the Risk-Need-Responsivity Framework

The three SAPROF subscales represent underlying concepts that can be arranged into a protective factor profile identifying patterns of strength and areas requiring attention in treatment. The SAPROF profile can also offer guidance for developing preventive measures and intervention plans. The risk-need-responsivity (or RNR) model of effective correctional treatment provides a framework for integrating assessment and intervention approaches to reduce risk and prevent recidivism (Andrews & Bonta, 2010; Bonta & Andrews, 2024). The risk principle states that service intensity should be matched to client risk level and that services should prioritize moderate and high-risk cases. Applied to the SAPROF, high risk clients likely have the fewest protective factors and the most to gain by developing strengths and resiliencies through appropriate dosage of services. The need principle states that dynamic risk factors (*aka* criminogenic needs) linked to crime and violence should be prioritized for treatment by risk reducing interventions. In the context of the SAPROF, the need principle would assert that a protective factor profile identifying areas of low strength across the Internal (e.g., poor self-control), Motivational (e.g., weak work ethic, problematic attitudes toward authority), and External (e.g., lack of services and supports) domains should be prioritized for services by way of developing prosocial skills and strategies to strengthen protective factor domains.

Finally, the responsivity principle states that service delivery should use cognitive behavioral methods of change and clinical skill to engage clientele (general responsivity) and to adapt services to unique client features to promote treatment retention and gain (specific responsivity). Applied to the SAPROF, certain client specific responsivity characteristics may have implications for the presence and growth of protective factors (e.g., motivation, education, work history items), as would other general responsivity factors (e.g., building a sound working alliance to maximize treatment retention and gain).

## Ethnocultural Context of Protective Factors

The SAPROF was developed in the Netherlands and has since been imported to more than a dozen countries and incorporated into service delivery within correctional and forensic mental health systems worldwide. Accordingly, much of the aforementioned lines of research could be construed as cross-cultural validations of the tool, as SAPROF ratings have demonstrated predictive validity for nonrecidivism in samples from Canada (Coupland & Olver, 2020), Australia (Klepfisz et al., 2024), New Zealand (Nolan et al., 2022), Austria (Yoon et al., 2018), and Japan (Kashiwagi et al., 2018), among others. However, many of the samples in these studies have been predominantly of White European ancestry, with Japan being a notable exception, or if ethnocultural diversity did exist in the samples, the psychometric properties of SAPROF ratings were examined on the aggregate sample. As such, little is currently known about the extent to which the psychometric properties extend to ethnocultural minority groups such as Black, Indigenous, and Other Peoples of Color (BIPOC). This is particularly salient given that in some countries, the use of forensic assessment measures has come under increasing legal scrutiny about their psychometric and cultural appropriateness with ethnocultural minority groups who tend to be overrepresented in correctional systems, such as Indigenous persons (Ewert *v*. Canada, 2015; Canada *v*. Ewert, 2016; Ewert *v*. Canada, 2018). Although recent research has marshaled support for the predictive properties of mainstream forensic assessment tools for recidivism in Indigenous correctional samples (Olver et al., 2024), the structural and convergent properties of these measures with BIPOC groups in general, and Indigenous persons specifically, has been similarly underexplored.

## Rationale for the Current Study and Hypotheses

The predictive validity of structured forensic risk assessment tool ratings for recidivism and related outcomes is a cardinal set of psychometric properties for these instruments; but predictive validity hinges on the broader construct validity (and reliability) of inferences made on the basis of test scores, which have important clinical, conceptual, and theoretical implications (Olver, Neumann, et al., 2018). This includes, but is not limited to, other indicators of construct validity (Cronbach & Meehl, 1955), specifically: (1) convergent validity, the degree to which measurements of a given construct that are theoretically or conceptually linked are correlated; (2) discriminant validity, representing a weak or marginal association between theoretically unrelated measures; and (3) factorial validity, referring to the extent to which test item scores can be grouped into theoretically or conceptually meaningful domains of item constellations (i.e., latent factors), representing the latent structural foundations of the instrument. Although the predictive properties of SAPROF ratings are well-researched (Burghart et al., 2023), research has yet to conduct a standalone examination of the structural properties of the tool, while limited research has examined its convergent properties. Even less research has examined SAPROF scores when administered at two or more timepoints, that is, dynamic protection.

The measurement instruments used in psychological assessments are imperfect as they provide structured operationalizations to measure latent (unobservable) concepts (Clark & Watson, 1995), and this includes the SAPROF. For instance, the three subscales of the SAPROF are item groupings that make good sense conceptually; however, these are rationally derived item constellations and little is known whether the subscale item groupings reflect the underlying latent structure of item scores on the instrument. Accordingly, one means is to empirically examine the three-subscale structure of the SAPROF to inform to what degree the tool measures the latent constructs it purports to measure, and if SAPROF ratings converge with measures of clinically and theoretically relevant constructs that may reinforce and extend the use of the tool in clinical and forensic practice. Why does this matter?

Formally examining the factorial and convergent validity of SAPROF ratings has theoretical and clinical value that may carry implications for its use in forensic risk assessment and treatment planning. Establishing factorial validity ensures that the items within a given tool are measuring distinct aspects of risk or protection in a coherent and consistent manner and that items within a given factor are tapping meaningful dimensions. The process also assists in identifying potential discrepancies in the factor structure, which can be modified to refine and strengthen the utility of the tool (Floyd & Widaman, 1995). Further, demonstrating that test scores converge with those from established measures indicates that the tool is accurately capturing its purported constructs. Finally, examination of SAPROF change scores can provide insight into the nature and meaning of therapeutic processes that may promote the development of protective factors.

The present study aimed to examine the structural and convergent properties of SAPROF scores in a large sample of treated men with sexual offense conviction histories with the SAPROF rated at two timepoints (i.e., pre and posttreatment). In the present investigation, factorial and convergent validity were examined by performing the following: exploratory and confirmatory factor analyses (EFA and CFA, respectively) to evaluate the best-fitting factor structure of the SAPROF relative to the data, followed by Exploratory Structural Equation Modeling (ESEM) to test the temporal stability of SAPROF ratings across repeated administrative time points (pre- and posttreatment). Convergent validity was then examined by way of examining correlations with psychometric measures of key psychological constructs indicative of risk/need (e.g., sexual violence risk, criminogenic need, and risk change) and general/specific responsivity (e.g., cognitive functioning, literacy, working alliance), per the RNR framework.

The study had five hypotheses. First, we predicted the data would generate a three-factor structure with at least some conceptual overlap with the original three-subscale structure of the SAPROF, corresponding to the Internal, Motivational, and External domains per de Vogel et al. (2011). Second, we anticipated that factor loadings would show temporal stability across pre- and posttreatment time points; specifically, that instrument scores retain their latent structure across repeated administrations, and that changes in item ratings (e.g., with treatment), do not alter the latent structure of test scores. Third, we anticipated SAPROF ratings to converge with measures of risk and need, specifically, to correlate negatively with measures of static and dynamic risk factors but that that pre–post changes on the SAPROF (corresponding to an increase in protection) will be positively correlated with treatment-related decreases in sexual violence risk and general criminal attitude assessed at the same timepoints. Fourth, we predicted that the SAPROF ratings would correlate positively with responsivity indictors that would bode well for engagement and reintegration such as the strength of the working alliance (general responsivity), and measures of cognitive functioning, work history, and education (specific responsivity). For instance, protective effects are considered to be evident when individuals have intact cognitive functioning (average or better); thus it stands to reason that measures of cognitive functioning and indicators of education would converge with other domains on the SAPROF (Coupland & Olver, 2020; Kennedy-Turner et al., 2020; Nikulina & Widom, 2019; Olver et al., 2011; Porter et al., 2023; Ttofi et al., 2016). Fifth, given prior research that has demonstrated important continuities in the predictive and structural properties of forensic assessment tools with Indigenous correctional populations (Olver et al., 2024), we anticipated that SAPROF ratings would demonstrate concordance in their structural and convergent properties across Indigenous and non-Indigenous (White majority) groups.

## Method

The present study utilized data from a broader investigation of protective factors, sexual violence risk, and change (Olver & Riemer, 2021) that received ethical approval from the University of Saskatchewan Behavioral Research Ethics Board (Beh # 15-366) and operational approval from Correctional Service Canada (CSC). Per Simmons et al. (2012), “We report how we determined our sample size, all data exclusions, all manipulations, and all measures in the study.”

### Participants

The study involved 461 men serving federal sentences who participated in sexual-offense treatment programs across a 10-year period (1998–2008) as a component of their rehabilitation plans through CSC. All participants were serving a sentence of at least 2 years with the average fixed sentence length being 4.9 years (*SD* = 3.0, *n* = 386); all of the men had either currently or previously been convicted for a sexually motivated offense. The majority (60.1%, 265/434) of the sample held a previous criminal conviction or charge for sexually offending. Roughly equivalent proportions had sexually offended solely against adults (45.9%, 199/ 434) or youth (41.7%, 181/434), whereas a minority (12.4%, 54/434) sexually offended against both adults and youth. At the time of sentencing for the men’s index offense(s), they were an average age of 36.2 years (*SD* = 11.8, *n* = 382) and an average age of 40.4 years (*SD* = 12.0, *n* = 432) at time of release. Men who self-identified as Indigenous ancestry (42.3%, 198/465) and White males (48.0%, 223/465) made up approximately equal shares of the sample, with the remaining men (9.5%, 44/465) classified as other/unknown. A minority were single/never married (37.9%, 159/420) with the majority having been ever married or equivalent (62.1%, 261/420).

### Sexual Offense Treatment Program

All of the men participated in sexual-offense treatment programs (SOTPs) either through the National Sex Offender Program (NaSOP) administered by the CSC, or alternatively, a comparable CSC-based high-intensity sexual offending program offered at a maximum-security correctional mental health facility, akin in content and structure to the high-intensity stream of the NaSOP. The majority of men were enrolled in high-intensity treatment programs with a duration of 8–9 months (*n* = 325/449), while the remainder were enrolled in prison-based streams of either moderate (4–5-month duration, *n* = 49/449) or low intensity (2 months, *n* = 75/449). The treatment programs were delivered under the direction of a registered psychologist and were cognitive behaviorally based, integrating the RNR principles. Given that the mandate of such programs was the reduction of sexual violence risk, treatment foci were invariably risk based, as opposed to strength based (e.g., Good Lives Model; Ward et al., 2007), but involved the development of prosocial skills, strategies, competencies, and supports that readily translated into increasing the strength and presence of protective factors. The facilitators of the treatment programs consisted of specialists from various health professions (e.g., occupational and recreational therapy, addictions, social work, and nursing); psychiatrists and nursing personnel oversaw medication regimes. Healthy sexuality and sexual self-regulation, interpersonal relationship and intimacy skills, regulation of emotions and anger management, alternatives to violent behavior, and recognizing and modifying attitudes and cognitions that reinforce sexual offending were among the common treatment targets; these modules were typically provided in a group setting and augmented by individual therapy. The majority of institutions provided supplementary programs including chemical dependency treatment, vocational and educational enhancement, along with cultural support and intervention services such as Indigenous healing, to complement the primary sexual offense treatment.

### Measures

#### The Structured Assessment of Protective Factors

The SAPROF (de Vogel et al., 2009, 2011) is a structured professional judgment (SPJ) tool intended to be used in conjunction with an established risk-assessment tool for case management, intervention planning, and risk assessment (Coupland & Olver, 2020; de Vogel et al., 2011; de Vries Robbé, de Vogel, et al., 2015; de Vries Robbé et al., 2013). The SAPROF consists of 17 protective items organized into 2 static (intelligence and secure childhood attachment) and 15 dynamic items, each rated on a 3-point scale of 0 (absence of a protective factor), 1 (partially present), and 2 (complete presence of protective factor). Item ratings can be summed mechanically to generate a final score ranging from 0 to 34 or examined configurally using professional judgment to generate a summary protection rating; higher scores/professional judgment ratings indicate a greater number of protective factors. The items are organized into three domains: Internal (i.e., intelligence, secure childhood attachment, empathy, coping, self-control), Motivational (i.e., work, leisure, financial management, motivation for treatment, attitude toward authority, life goals), and External (i.e., social network, intimate relationship, professional care, living circumstances, external control). Several lines of validation research support the use of comprehensive file information to obtain quality SAPROF ratings (Coupland & Olver, 2020; de Vries Robbé et al., 2013; de Vries Robbé, de Vogel, et al., 2015; Yoon et al., 2018). Good to excellent interrater agreement was obtained per the Cicchetti and Sparrow (1981) guidelines via intraclass correlation coefficient (ICC; one-way random effects model, single measure, absolute agreement) for SAPROF ratings on 32 independently double-coded randomly selected protocols: SAPROF total (pre) ICC_A1_ = .71, (post) ICC_A1_ = .76, (change) ICC_A1_ = .70. SAPROF total scores demonstrated good internal consistency (Cronbach’s α): pre = .82, post = .83.

#### Risk and Need Measures

##### Static-99R

The Static-99R is an actuarial risk assessment tool designed to predict sexual recidivism (Helmus et al., 2012). The tool consists of 10 static items, reflecting sexual and nonsexual offense history as well as victim demographics. Total sores range from –3 to 12 and correspond to risk categories reflective of the five-level system: Level I (very low risk, –3, –2), Level II (below average risk, –1, 0), Level III (average risk, 1–3), Level IVa (above average risk, 4, 5), and Level IVb (well above average risk, 6–12). Meta-analytic support from 8,390 sexually offending persons and 24 samples demonstrated good predictive accuracy for sexual recidivism (AUC = .72; Helmus et al., 2012).

##### Violence Risk Scale-Sexual Offense Version

The VRS-SO is an established sexual offense risk assessment and treatment planning tool developed in 2003 (Wong et al., 2003; Wong, 2016). The tool consists of 7 static (e.g., age at time of release, age at first sexual offense, sexual offense victim profile) and 17 dynamic (e.g., sexually deviant lifestyle, interpersonal aggression, cognitive distortions) items organized into three domains demonstrated by factor analysis: Sexual Deviance (e.g., sexually deviant lifestyle, deviant sexual preference), Criminality (e.g., interpersonal aggression, substance abuse, impulsivity), and Treatment Responsivity (e.g., cognitive distortions, lack of insight, poor treatment compliance; Olver et al., 2007). Each item is rated on a 4-point scale ranging from 0 (no significant relationship between the factor and sexual offending), 1 (less positive than a 0), 2 (less serious or negative than a 3), and 3 (a significant relationship between the factor and sexual offending). The weight and versatility of offending history is reflected in higher ratings on the static items. Typically, for dynamic ratings, the higher the rating, the more the factor is associated with sexual offending, and therefore, prioritized as targets for treatment. Using a modified version of Prochaska et al.’s (1992) transtheoretical model of change, the VRS-SO evaluates change on its 17 dynamic items. The five stages of change (Pre-contemplation, Contemplation, Preparation, Action, and Maintenance) are operationalized for each dynamic item. Dynamic items scored as a 0 or 1 are similarly not considered treatment targets, and therefore, not given a stages-of-change rating. Treatment targets (dynamic items scored as a 2 or 3) are given a stages-of-change rating pre-treatment and then re-rated posttreatment to determine treatment progress. Progression from one stage to the next stage is indicative of positive change, and accordingly, risk reduction is scored as a 0.5-point reduction in the pre-treatment rating of the item; progression of two stages, a 1.0-point reduction; and so on. In contrast, deterioration in between stages can be measured through a corresponding increase in score. To determine a posttreatment score for the dynamic items, this process is completed for each item that has been designated as a treatment target.

##### Criminal Sentiments Scale

The Criminal Sentiments Scale (CSS; Gendreau et al., 1979) is a self-report 41-item measure designed to assess criminal attitudes organized into three sub-scales, Law, Court, and Police (LCP), Tolerance Law Violation (TLV), and Identification with Criminal Others (ICO). The CSS is rated on 5-point Likert-type scale (–2 = *strongly disagree/agree* to 2 = *strongly agree/disagree*), with some items reverse scored. The LCP subscale comprises 25 items regarding adversarial attitudes toward the law, courts, and police. The TLV subscale consists of 10 items that condone offending behavior. The ICO subscale includes 6 items reflecting similarity to individuals who break the law. Items from the LCP scale are scored so that higher scores indicate positive attitudes toward the law, courts and police. In contrast, high scores on the TLV and ICO scales represent pro-criminal attitudes. Witte et al. (2006) reported high overall internal consistency (α = 0.94) of the CSS and moderate to high predictive validity for non-violent and violent recidivism with a sexually offending sample.

#### Responsivity Measures

##### Working Alliance Inventory

The Working Alliance Inventory (WAI) is a self-report 36-item measure regarding the strength and quality of the therapeutic relationship between the client and therapist (aka therapeutic alliance; Horvath & Greenberg, 1989). Each item (e.g., I am worried about the outcome of these sessions, I am clear on what my responsibilities are in therapy) is rated on a 7-point Likert-type scale ranging from 1 (Never) to 7 (Always). Scores can range from 36 to 252 with higher scores reflecting a stronger working alliance. A total score can be obtained by adding up the items, which can also be arranged into three 12-item subscales measuring Task (i.e., how much the sessions are defined by relevant tasks or interventions), Bond (i.e., the degree to which the client and therapist feel a warm, empathic emotional connection), and Goal (i.e., to what extent the client and therapist share common goals and expected treatment outcomes). Previous research from Olver et al. (2023) demonstrated that in a sample of 317 treated men, the WAI subscales were highly intercorrelated (all *p* < .001): Task × Bond *r* = .70; Bond × Goal *r* = .74; and Task × Goal *r* = .86.

##### Cognitive Functioning and Literacy Measures

Scores on multiple measures of cognitive functioning were examined in the present sample. Research suggests that lower levels of cognitive ability are associated with increased risk of engaging in violent and antisocial behavior (Frisell et al., 2012; Hirschi & Hindelang, 1977). Conversely, higher levels of cognitive ability have been associated with a mitigation in offending behavior (Nikulina & Widom, 2019; Ttofi et al., 2016). Nikulina and Widom (2019) found higher scores on measures assessing verbal intelligence, reading ability, cognitive flexibility, and nonverbal reasoning, demonstrated protective associations with future violent criminal activity. Four measures were employed: (1) *Canadian Adult Achievement Test (CAAT) Reading Comprehension* (Fowler, 1997; Muirhead & Rhodes, 1998): a measure of reading comprehension comprised of 50 multiple-choice questions focused on comprehension, inference-making, and drawing conclusions from passages of a functional and educational nature (Fowler, 1997); (2) *Raven’s Progressive Matrices*: a non-verbal, 60-item multiple-choice intelligence test of abstract reasoning and general cognitive ability (Raven, 2000). The measure presents a series of diagrams or designs with a missing part, requiring individuals to select the correct completion; (3) *Symbol Digit Modalities Test (SDMT)*: originally developed in 1973 as a measure of processing and motor speed (Kiely et al., 2014; Smith, 1973) and concordant functions of attention, visual scanning, tracking, and working memory. The SDMT requires individuals to identify nine symbols paired with a number 1 through 9, then correctly manually inputting corresponding numbers beneath a row of symbols; (4) *Quick Test* (QT): a rapid screening tool designed to measure verbal-perceptual intelligence (Ammons & Ammons, 1962) comprising three single forms containing 50-word items.

##### Employment and Education

Research has demonstrated poor work history and low education to be risk factors but their alternatives have protective effects including increased correctional treatment completion and reduced recidivism (Coupland & Olver, 2020; Kennedy-Turner et al., 2020; Olver et al., 2011; Porter et al., 2023). Binary employment history of stable (i.e., regular employment or at least employed more than 6 months during the year prior to sentence) versus unstable (i.e., never employed, frequently unemployed, or unemployed more than 6 months prior to current sentence) and self-reported years of education were examined as convergent measures.

### Procedure

The present sample was retrieved via a digital database developed from a wider examination that investigated sexual violence risk, treatment change, and recidivism featuring the VRS-SO and related measures (Olver, Mundt et al., 2018). The self-report measures (CSS, WAI, SDMT, Ravens, Quick Test) were completed by the men and administered as part of routine services during the men’s program attendance by a research psychometrist. A subsequent investigation using this participant sample rated structured measures of psychopathy and protective factors, through file review, and examined links to posttreatment community recidivism (Olver & Riemer, 2021); no interviews had been conducted. A large literature supports the validity and reliability of file-based ratings for major forensic assessment tools, including assessments of psychopathy (e.g., Harris et al., 2013; Wong, 1988), dynamic risk factors (e.g., Coupland & Olver, 2020; Douglas et al., 2005), and protective factors (e.g., Coupland & Olver, 2020; de Vries Robbé, de Vogel et al., 2015). Personnel with security-clearance from the CSC accessed the men’s files via the Offender Management System. For each case, the documents had been archived digitally in distinct pre- and posttreatment folders. No direct involvement in coding the study’s measures was undertaken by the personnel who accessed the files and extracted the documents. The men’s files were comprehensive and often voluminous. They had participated in SOTPs and had thorough records regarding their behavior and advancements throughout treatment including an intake summary, interim report, and concluding treatment report. Moreover, when available, casework notes, criminal profile reports, psychiatric discharge summaries, psychological assessment reports, correctional plans, and other significant decision documentation were accessed.

To complete pretreatment SAPROF ratings, pretreatment details were gathered from all sources between the time of admission for the index sentence and the time of the intake summary (which was completed shortly thereafter admission to the SOTP). To complete posttreatment SAPROF ratings, posttreatment data were exclusively collected from interim and final treatment progress assessments and other notable documents prepared at the conclusion of the program and preceding community release. The timeframe during which the SAPROF ratings were completed, while the men were still in custody, enabled eliminating potential reverse causation; that is, posttreatment SAPROF scores would not indicate a reduction of protective factors as a result of recidivism; instead, the scores indicate the extent to which these were potentially altered throughout the pre–posttreatment period, and specifically, prior to release. The RAs generally did not have access to data on any of the study measures, as these were either contained in a hardcopy psychometric treatment file or were completed independently by other raters as separate parts of the larger investigation. An exception was that about one-third of the files (from low-intensity and high-intensity programs offered at two of the institutions) had VRS-SO ratings completed by service providers written into the pre and posttreatment program reports.

The research team included seven senior undergraduate psychology students from the host institution who were trained on the SAPROF by the principal investigator (PI), a registered psychologist with 20 years of research and clinical practice in correctional settings. Each student researcher had successfully completed coursework in forensic psychology and either completed or were close to completing their honors degree. All research assistants (RAs) first completed practice ratings on redacted training cases and later co-coded the inaugural 5 files. This was followed by separately double-coding 32 files to assess interrater reliability (IRR). Initially, 20 cases were randomly chosen and assigned for IRR coding and throughout the process of accumulating the data, additional files would be periodically and randomly selected (*n* = 12) for IRR coding to avoid rater drift. Precision inspections and consultation regarding case ratings were additionally provided, and the PI provided a refresher training on the study’s measures roughly a year into the study. All SAPROF data were entered into a spreadsheet by a senior RA that was cross-checked by the PI for accuracy.

### Data Analyses

Several analyses were conducted to examine the structural and convergent validity of SAPROF scores. All factor analyses utilized Mplus v. 8.10 (Muthén & Muthén, 2023), while SPSS v.28 was employed for convergent validity analyses. In his review of power considerations for factor analyses, Kyriazos (2018) notes that, although factor analysis is a large sample technique, smaller sample sizes can be employed with better quality data such as items with high factor loadings, few cross loadings, large communalities, and five or more items per factor. In lieu of conducting formal power analyses, we heeded these robust data property conventions that indicate sufficient analytic power and stability of the resultant solution. Further, Kyriazos (2018) adds that a subjects-to-variable ratio of 5:1 for EFA and 10:1 to 20:1 for CFA/SEM is a common guideline; here, the total *N* of 414 and ethnocultural subgroup *n*s (non-Indigenous *n* = 219; Indigenous *n* = 183) for factor analysis handily met or exceeded these conventions. Taken together, we concluded that the SAPROF data properties and sample size indicated adequate power to execute the following sets of analyses.

For all factor analyses, which involve examination of ordinal (i.e., ordered categorical) scale items, default Mplus settings were employed which consisted of weighted least squares means and variance adjusted (WLSMV) model estimation and Geomin oblique factor rotation. The default WLSMV model estimation procedure analyzes a polychoric correlation matrix for categorical variables, in contrast to the Pearson correlations used in other factor analytic model estimation procedures (e.g., maximum likelihood) which apply to continuous variables. Moreover, with ordered categorical variables, polychoric correlations provide a more accurate estimate of the association between variables when they are assumed to be indicators representing underlying normally distributed continuous variables (Kiwanuka et al., 2022). Given that EFA aims to identify the nature and number of latent constructs underlying a set of observed variables (Omura et al., 2022), we began with conducting an EFA on 16 SAPROF items (omitting item 12, medication adherence) to identify the best fitting factor structure for the given data. Medication adherence is the only SAPROF item with the option of being rated as not applicable (n/a) and rating guidelines identify that it be omitted when it does not apply (de Vogel et al., 2009). Given that this item had n/a ratings for the large majority of cases, it was excluded from factor analyses. We began with an EFA to evaluate the structural viability of the three-subscale (factor) model and then followed with CFA, first testing the original SAPROF structure and then one informed by the results of EFA, per L. Muthén (2004) recommendations to start “with an EFA to weed out bad items and factors, then do an EFA in a CFA framework to obtain standard errors for the factor loadings, and then do a simple structure CFA” (Mplus Discussion Board).

For the CFA, Santor et al. (2011) note these provide valuable information about how well the data fits a particular theory-derived measurement model (i.e., items only load on the components they are intended to measure) and as highlighting potential weaknesses of specific items. Accordingly, a CFA was conducted to determine if the original three subscale model is similar to the best-fitting model identified by the EFA. Thus, the CFA was able to test which observed variables are related to the specified latent factors.

Marsh et al. (2010) argue that CFAs may be too restrictive and propose that ESEM is an innovative technique for factor analysis that integrates EFA and CFA approaches and has broad applicability to various fields of psychology based on the assessment of latent constructs. The ESEM approach assesses measurement invariance which refers to the mean level-difference across multiple time points (e.g., observing the same sample over pre and posttreatment time-points) or groups (e.g., age groups), the former is of particular importance to the present study (Marsh et al., 2014). Measurement invariance identifies if underlying factor structures are analogous across different time points or groups. For instance, Babchishin (2013) used ESEM to demonstrate temporal stability for the factor structures of the Acute 2007 (a dynamic risk assessment measure) across three administrative time points. As such, ESEM was employed to establish the best fitting latent structure and the stability of loadings across the two time points (pre- and posttreatment). The factor analyses concluded with an EFA conducted on SAPROF pre–post change scores to evaluate the latent structure of changes in protective factors with treatment; we did not have a priori hypotheses for these analyses, but the results would speak to the structure of change dimensions of this instrument in a correctional treatment program. Model fit across all factor analyses (EFA, CFA, ESEM) were examined via comparative fit index (CFI) and root mean squared error of approximation (RMSEA)—as a heuristic, CFI values of .90 to .95 and RMSEA values at or below .08 represent acceptable model fit to the data (Marsh et al., 2010).

To cross-validate the factor analytic results for SAPROF item ratings, we conducted multi-group ESEM (MG-ESEM) to examine the measurement invariance of pre and posttreatment SAPROF ratings across Indigenous and non-Indigenous ethnoracial groups. ESEM was then used to examine the latent structure of SAPROF change ratings within Indigenous and non-Indigenous ethnocultural groups. We finished with a set of convergent validity analyses to examine the risk, need, and responsivity relevance of SAPROF ratings by examining associations between SAPROF scores and conceptually relevant measures in a multitrait-multimethod matrix. We used Cohen’s (1992) conventions for interpreting correlation magnitudes between two continuous variables: small (*r* = .10), medium (*r* = .30), and large (*r* = .50). For consistency and transparency, convergent validity analyses were completed both on the aggregate sample and across ethnocultural subgroups, power limitations notwithstanding.

## Results

### Factor Analyses and Exploratory Structural Equation Modeling of SAPROF Item Scores

Complete pre- and posttreatment item scores were available for 414 cases to conduct CFA and ESEM analyses. An initial CFA of the original three-factor model did not meet conventional thresholds for a good fit, WLSMV χ^2^ (101) = 739.315, CFI = .839, RMSEA = .124, 90%CI = [.116, .133], for the inaugural three-factor subscale. The results of an EFA demonstrated that the data fit a different three-factor structure, yielding acceptable fit statistics for both sets of ratings at pretreatment, WLSMV χ^2^ (75) = 192.494, CFI = .970, RMSEA = .062, 90%CI = [.051, .073], and posttreatment, WLSMV χ^2^ (75) = 174.411, CFI = .985, RMSEA = .057, 90%CI = [.046, .068], with some item loadings diverging from the original subscale structure. A modified CFA demonstrated good fit, WLSMV χ^2^ (101) = 318.271, CFI = .945, RMSEA = .073, 90%CI = [.064, .082], and improved considerably in comparison to the original three-factor subscale. A modified CFA of this model demonstrated good fit, WLSMV χ^2^ (101) = 318.271, CFI = .945, RMSEA = .073, 90%CI = [.064, .082], and improved considerably in comparison to the original three-factor subscale.

The results of ESEM of pre and post SAPROF item ratings demonstrated excellent model fit, WLSMV χ^2^ (420) = 750.658, CFI = .978, RMSEA = .044, 90%CI = [.039, .049]. Standardized factor loadings for pre and posttreatment ratings for the ESEM are reported in Table 1. The SAPROF Internal factor was not replicated in its entirety, comprising three of its original items (3, 4, and 5), and picking up three additional items (9, 10, and 11) originally from the Motivational subscale; for the present sample, it was labeled Internal-Prosocial. The second factor, Motivational, also retained three of its original items (6, 7, and 8) with items 1, 2, 13, and 14 additionally loading; it was relabeled Motivational-Lifestyle. The third factor, External, most closely paralleled its original factor fit and was limited to three items (15, 16, and 17) that loaded highly and exclusively on this factor, and retaining its original label. The results further demonstrated temporal stability in factor loading magnitudes across pre and post time points (Table 1). The analyses above were cross-validated through conducting MG-ESEM of SAPROF pre and posttreatment ratings (see Table 2). The same three correlated factors—Internal-Prosocial, Motivational-Lifestyle, and External—were reproduced in their entirety across Indigenous and non-Indigenous ethnocultural groups and the two timepoints with excellent fit: CFI = .987, RMSEA = .039, 90%CI = [.033, .045].

**Table 1.** SAPROF Loadings for Pre- and Posttreatment Item Ratings From Exploratory Structural Equation Modeling.

SAPROF items	Internal (prosocial)		Motivational (lifestyle)		External	
	Pre	Post	Pre	Post	Pre	Post
	Λ (*SE*)	Λ (*SE*)	Λ (*SE*)	Λ (*SE*)	Λ (*SE*)	Λ (*SE*)
1 Intelligence	.140 (.072)	.160 (.082)	**.350** (.080)	**.326** (.075)	.294 (.070)	.262 (.061)
2 Secure attachment in childhood	–.003 (.062)	–.003 (.070)	**.510** (.074)	**.475** (.070)	.157 (.071)	.140 (.063)
3 Empathy	**.686** (.044)	**.783** (.047)	–.041 (.068)	–.038 (.063)	.048 (.055)	.043 (.049)
4 Coping	**.794** (.026)	**.907** (.022)	–.012 (.023)	–.011 (.022)	.145 (.050)	.129 (.044)
5 Self Control	**.667** (.048)	**.761** (.052)	.026 (.040)	.024 (.038)	.363 (.050)	.324 (.043)
6 Work	.230 (.068)	.262 (.078)	**.584** (.071)	**.544** (.071)	.106 (.084)	.094 (.075)
7 Leisure activities	.210 (.079)	.240 (.090)	**.468** (.076)	**.437** (.075)	–.156 (.067)	–.139 (.060)
8 Financial management	–.013 (.035)	–.014 (.040)	**.832** (.053)	**.775** (.049)	.153 (.106)	.137 (.094)
9 Motivation for treatment	**.898** (.049)	**1.024** (.051)	–.188 (.077)	–.175 (.071)	–.004 (.007)	–.004 (.006)
10 Attitude toward authority	**.747** (.044)	**.853** (.047)	.074 (.069)	.069 (.064)	.033 (.042)	.030 (.038)
11 Life goals	**.628** (.057)	**.716** (.064)	.239 (.069)	.223 (.065)	–.122 (.052)	–.109 (.046)
13 Social network	.290 (.067)	.331 (.077)	**.512** (.064)	**.478** (.065)	–.014 (.016)	–.013 (.015)
14 Intimate relationship	.092 (.100)	.105 (.114)	**.486** (.092)	**.453** (.088)	–.191 (.075)	–.171 (.067)
15 Professional care	.185 (.079)	.211 (.090)	–.059 (.060)	–.055 (.056)	**.770** (.031)	**.687** (.030)
16 Living circumstances	–.013 (.012)	–.014 (.013)	.318 (.075)	.297 (.071)	**.694** (.043)	**.619** (.038)
17 External control	.031 (.091)	.036 (.104)	.018 (.030)	.017 (.028)	**1.015** (.036)	**.906** (.034)
Eigenvalue	6.387	6.723	2.249	2.243	1.401	1.408

*Note: N* = 414. Factor loadings designating an item to load on a given factor are in bold font. Item 12 N/A.

**Table 2.** Multigroup Exploratory Structural Equation Modeling (MG-ESEM): SAPROF Loadings for Pre- and Posttreatment Item Ratings Across Indigenous and Non-Indigenous Subgroups.

SAPROF items	Internal (prosocial)				Motivational (lifestyle)				External			
	Pre (Λ, *SE*)		Post (Λ, *SE*)		Pre (Λ, *SE*)		Post (Λ, *SE*)		Pre (Λ, *SE*)		Post (Λ, *SE*)	
	NI	I	NI	I	NI	I	NI	I	NI	I	NI	I
1 Intelligence	.031 (.085)	.042 (.114)	.036 (.099)	.060 (.164)	**.444 (.100)**	**.444 (.112)**	**.427 (.103)**	**.497 (.145)**	.229 (.056)	.278 (.063)	.219 (.052)	.396 (.082)
2 Secure attachment in childhood	–.088 (.116)	–.097 (.127)	–.102 (.134)	–.115 (.152)	**.619 (.121)**	**.512 (.117)**	**.596 (.122)**	**.466 (.126)**	.028 (.070)	.028 (.070)	.027 (.066)	.033 (.081)
3 Empathy	**.666 (.051)**	**.670 (.072)**	**.771 (.056)**	**.777 (.057)**	–.006 (.066)	–.004 (.050)	–.005 (.063)	–.004 (.044)	.042 (.051)	.038 (.047)	.040 (.049)	.044 (.053)
4 Coping	**.796 (.038)**	**.890 (.055)**	**.921 (.032)**	**.931 (.037)**	–.004 (.030)	–.004 (.025)	–.004 (.028)	–.003 (.020)	.115 (.047)	.117 (.048)	.109 (.044)	.120 (.048)
5 Self Control	**.684 (.059)**	**.750 (.083)**	**.791 (.065)**	**.865 (.075)**	.028 (.062)	.023 (.051)	.027 (.060)	.020 (.046)	.307 (.051)	.307 (.055)	.293 (.045)	.346 (.057)
6 Work	.058 (.120)	.076 (.158)	.068 (.139)	.088 (.179)	**.730 (.103)**	**.718 (.126)**	**.703 (.110)**	**.635 (.143)**	–.005 (.039)	–.007 (.046)	–.005 (.037)	–.007 (.052)
7 Leisure activities	.143 (.135)	.118 (.113)	.165 (.157)	.137 (.129)	**.605 (.103)**	**.378 (.097)**	**.582 (.109)**	**.336 (.094)**	–.238 (.073)	–.180 (.062)	–.228 (.072)	–.204 (.071)
8 Financial management	–.144 (.145)	–.193 (.195)	–.166 (.168)	–.254 (.259)	**.894 (.117)**	**.904 (.160)**	**.861 (.127)**	**.917 (.191)**	.033 (.065)	.040 (.081)	.031 (.062)	.052 (.103)
9 Motivation for treatment	**.903 (.072)**	**.961 (.077)**	**1.045(.074)**	**.997 (.064)**	–.208 (.094)	–.166 (.076)	–.200 (.090)	–.133 (.061)	.002 (.012)	.002 (.011)	.002 (.011)	.002 (.012)
10 Attitude toward authority	**.705 (.062)**	**.759 (.069)**	**.816 (.067)**	**.890 (.060)**	.100 (.082)	.081 (.068)	.097 (.079)	.073 (.062)	.010 (.033)	.010 (.032)	.010 (.031)	.011 (.037)
11 Life goals	**.551 (.078)**	**.619 (.088)**	**.638 (.089)**	**.627 (.082)**	.276 (.076)	.233 (.072)	.265 (.076)	.182 (.061)	–.195 (.044)	–.199 (.048)	–.186 (.043)	–.198 (.047)
13 Social network	.176 (.106)	.207 (.124)	.204 (.124)	.234 (.140)	**.604 (.088)**	**.532 (.105)**	**.581 (.094)**	**.463 (.108)**	–.114 (.065)	–.122 (.068)	–.109 (.063)	–.135 (.079)
14 Intimate relationship	–.001 (.028)	–.001 (.041)	–.001 (.032)	–.001 (.035)	**.521 (.071)**	**.576 (.077)**	**.502 (.067)**	**.375 (.061)**	–.267 (.063)	–.359 (.094)	–.256 (.061)	–.297 (.075)
15 Professional care	.322 (.105)	.370 (.131)	.373 (.120)	.296 (.106)	–.081 (.074)	–.070(.065)	–.078 (.071)	–.043 (.041)	**.733 (.040)**	**.768 (.044)**	**.701 (.038)**	**.602 (.059)**
16 Living circumstances	–.008 (.006)	–.009 (.007)	–.009 (.007)	–.009 (.007)	.462 (.091)	.361(.085)	.445 (.090)	.299 (.076)	**.615 (.060)**	**.583 (.061)**	**.588 (.052)**	**.614 (.068)**
17 External control	.173 (.123)	.204 (.153)	.200 (.142)	.183 (.135)	.020 (.015)	.018(.014)	.019 (.015)	.012 (.010)	**.965 (.047)**	**1.035 (.050)**	**.922 (.044)**	**.915 (.053)**

*Note*: NI = non-Indigenous, *n* = 219; I = Indigenous, *n* = 183. Item loadings designated to a given factor are in bold font.

### Factor Analyses and Exploratory Structural Equation Modeling of SAPROF Change Ratings

An EFA of SAPROF change scores (excluding item 12 and the two ostensibly static items, 1 and 2) generated a two-factor model that provided a strong fit to the data: CFI = .951, RMSEA = .060, 90%CI = [.048, .073]. The two change factors were labeled: (1) Psychosocial, which had most items from the Internal and Motivational subscale, along with social network (item 13) from the External scale, loading, and (2) External, which comprised the last three items of the instrument from the External factor that similarly loaded on this factor across the EFA, CFA, and ESEM analyses (see Table 3). Change scores on finances (item 8) and intimate relationships (item 14) items did not load on either factor. The results of ESEM applied to SAPROF change scores generated substantively the same two change factors with acceptable fit across ethnocultural groups: Indigenous CFI = .912, RMSEA = .074 90%CI = [.054, .093], non-Indigenous CFI = .953, RMSEA = .060, 90%CI = [.040, .078]. For the Indigenous group, change scores for Item 8, financial management, also loaded on the psychosocial change factor, while for the non-Indigenous group, neither this item nor Item 6, work change, scores loaded on the factor. Both external change factors were identical to the original solution on the total sample (Table 3).

**Table 3.** Factor Loading Matrix for Exploratory Factor Analysis of SAPROF Item Change Scores (Total Sample) and Exploratory Structural Equation Modeling (ESEM) Results for Indigenous and Non-Indigenous Subgroups.

SAPROF Item (change score)	Total sample		Indigenous		Non-Indigenous	
	Psychosocial	External	Psychosocial	External	Psychosocial	External
	Λ (*SE*)	Λ (*SE*)	Λ (*SE*)	Λ (*SE*)	Λ (*SE*)	Λ (*SE*)
3. Empathy	**.634** (.043)	–.028 (.069)	**.678 (.071)**	–.040 (.126)	**.620 (.056)**	.003 (.058)
4. Coping	**.755** (.045)	–.064 (.085)	**.850 (.053)**	.012 (.081)	**.652 (.064)**	–.067 (.108)
5. Self-Control	**.654** (.038)	–.186 (.082)	**.667 (.102)**	–.197 (.118)	**.587 (.051)**	–.122 (.108)
6. Work	**.308** (.064)	.065 (.073)	**.490 (.106)**	–.126 (.122)	.201 (.094)	.098 (.087)
7. Leisure activities	**.512** (.062)	.094 (.081)	**.499 (.136)**	.204 (.121)	**.581 (.077)**	–.021 (.086)
8. Financial management	.107 (.091)	–.121 (.090)	**.320 (.355)**	–.582 (.197)	.067 (.111)	.009 (.100)
9. Motivation for treatment	**.619** (.036)	.191 (.054)	**.645 (.085)**	.122 (.118)	**.576 (.051)**	.249 (.064)
10. Attitude toward authority	**.647** (.043)	.002 (.006)	**.610 (.079)**	–.092 (.111)	**.690 (.058)**	.056 (.076)
11. Life goals	**.637** (.042)	.048 (.065)	**.545 (.113)**	.163 (.112)	**.725 (.050)**	–.004 (.044)
13. Social network	**.416** (.051)	.187 (.068)	**.326 (.186)**	.322 (.104)	**.500 (.066)**	.129 (.092)
14. Intimate relationship	.074 (.053)	.003 (.055)	.100 (.089)	.088 (.100)	.041 (.087)	–.152 (.076)
15. Professional care	.004 (.003)	**.876** (.038)	.063 (.402)	**.779 (.072)**	.003 (.002)	**.940 (.056)**
16. Living circumstances	.161 (.054)	**.564** (.040)	.116 (.292)	**.563 (.068)**	.214 (.077)	**.521 (.057)**
17. External control	–.115 (.047)	**.766** (.037)	–.078 (.410)	**.816 (.056)**	–.121 (.054)	**.775 (.048)**
Eigenvalue	3.775	2.175	3.879	2.362	3.722	2.215

*Note*: Total *N* = 383, item change scores loading on a given factor are bolded; Indigenous *n* = 174, non-Indigenous *n* = 200.

### Convergent Validity Analyses

Convergent validity analyses utilized scores generated from the SAPROF’s original three subscales and its scale total, such that results can be generalized to the structure of the instrument as it is currently used in research and practice. SAPROF ratings were correlated with scores from conceptually relevant risk/need and responsivity measures across pretreatment, posttreatment, and change ratings.

### Associations With Risk and Need Measures

Correlations between scores on the SAPROF measures and those on measures of static and dynamic sexual violence risk and criminal attitudes are reported in Table 4 and arranged by total sample (T), and Indigenous (I) and non-Indigenous (NI) subgroups. First, pretreatment ratings demonstrated several significant negative correlations, small to large in magnitude. Particularly strong negative correlations, medium to large in magnitude, were observed between SAPROF total pre ratings and the VRS-SO total and Criminality factor, as well as Static-99R ratings, both within the aggregate sample and across ethnocultural groups. Consistent with the pattern of findings for pretreatment ratings, correlations for posttreatment ratings also yielded moderate to large negative correlations. Second, SAPROF subscale ratings demonstrated moderate to large negative correlations with VRS-SO Criminality, Treatment Responsivity, and Dynamic total scores, while such associations tended to be smaller in magnitude and frequently nonsignificant with the Sexual Deviance factor; again, strong continuity in correlation magnitudes was observed across ethnocultural groups. Third, positive correlations that were broadly medium in magnitude were observed for change ratings for the SAPROF and VRS-SO dynamic total and factor scores, with the exception of the SAPROF’s External subscale. Finally, several significant small to medium negative correlations were observed between SAPROF and TLV and ICO scores across pre and post ratings and positive associations with the LCP subscale. By contrast, weaker convergent associations were found between the SAPROF External subscale and CSS measures, as well as for correlations between CSS and SAPROF change ratings. SAPROF-CSS convergent validity correlations also tended to be higher in the Indigenous group for LCP and TLV scores, but higher in the non-Indigenous group for ICO scores.

**Table 4:** Convergent Validity Correlations Between Scores on Risk/Need Convergent Measures With SAPROF Ratings as a Function of Indigenous Heritage.

Convergent risk/need measures		SAPROF measures											
	Group	Internal			Motivational			External			Total		
	T/NI/I	Pre	Post	Change	Pre	Post	Change	Pre	Post	Change	Pre	Post	Change
Static-99R	T	–.35**	–.29**	.02	–.47**	–.32**	.18**	–.24**	–.22**	.07	–.45**	–.36**	.15*
	I	–.34**	–.21*	.11	–.36**	–.27**	.08	–.18	–.16	.06	–.36**	–.28**	.11
	NI	–.33**	–.31**	–.04	–.51**	–.33**	.24**	–.25**	–.26**	.05	–.48**	–.38**	.16
VRS-SO													
Static	T	–.33**	–.27**	.03	–.40**	–.27**	.16**	–.22**	–.18**	.09	–.40**	–.31**	.15*
	I	–.34**	–.18	.15	–.33**	–.23*	.11	–.20*	–.14	.11	–.35**	–.24**	.17
	NI	–.30**	–.30**	–.05	–.41**	–.27**	.19*	–.21**	–.20*	.06	–.40**	–.32**	.13
Dynamic	T	–.37**	–.47**	.36**	–.40**	–.46**	.39**	–.29**	–.27	.15*	–.44**	–.52**	.41**
	I	–.44**	–.45**	.37**	–.41**	–.44**	.42**	–.26**	–.22*	.09	–.45**	–.48**	.41**
	NI	–.32**	–.48**	.35**	–.38**	–.47**	.38**	–.29**	–.31**	.22**	–.43**	–.55**	.44**
Total	T	–.40**	–.45**	-	–.44**	–.44**	-	–.29**	–.27**	-	–.47**	–.50**	-
	I	–.45**	–.39**	-	–.42**	–.41**	-	–.27**	–.22*	-	–.46**	–.44**	-
	NI	–.35**	–.47**	-	–.43**	–.45**	-	–.28**	–.31**	-	–.46**	–.52**	-
Sexual deviance	T	–.08	–.16**	.23**	–.06	–.15*	.22**	–.09	–.10	.09	–.09	–.17**	.25**
	I	–.15	–.13	.25**	–.07	–.11	.28**	–.10	–.09	.06	–.12	–.14	.28**
	NI	–.08	–.22**	.22**	–.10	–.22**	.22**	–.12	–.13	.14	–.13	–.25**	.27**
Criminality	T	–.39**	–.46**	.27**	–.48**	–.45**	.39**	–.30**	–.26**	.16**	–.50**	–.51**	.38**
	I	–.41**	–.47**	.32**	–.48**	–.50**	.40**	–.22*	–.22*	.10	–.46**	–.53**	.37**
	NI	–.34**	–.43**	.24**	–.45**	–.41**	.39**	–.31**	–.29**	.20*	–.48**	–.48**	.40**
Trt. responsivity	T	–.27**	–.41**	.33**	–.26**	–.43**	.31**	–.14*	–.20**	.12	–.28**	–.45**	.34**
	I	–.36**	–.40**	.32**	–.32**	–.41**	.32**	–.19*	–.13	.07	–.35**	–.42**	.32**
	NI	–.20*	–.41**	.35**	–.21**	–.44**	.31**	–.08	–.26**	.17*	–.21**	–.48**	.37**
CSS													
LCP	T	.21	.34**	.05	.30**	.33**	.10	.13	.08	–.16	.29**	.34**	.00
	I	.26	.33	.03	.42**	.40*	.27	.15	.09	–.13	.39*	.37*	.11
	NI	.09	.34	.09	.16	.26	–.05	.00	.02	–.15	.12	.28	–.08
TLV	T	–.20	–.34**	–.14	–.26*	–.38**	.01	–.14	–.18	.00	–.26*	–.41**	–.04
	I	–.24	–.41*	–.06	–.37*	–.47*	.18	–.20	–.34*	.09	.36*	.51**	.14
	NI	–.06	–.25	–.26	–.12	–.28	–.17	.00	–.01	–.01	–.09	–.25	–.18
ICO	T	–.28*	–.30*	–.01	–.26*	–.27*	.02	–.23*	–.08	.05	–.32**	–.30*	.04
	I	–.17	–.36	.12	–.15	–.29	–.10	–.29	–.35	.15	.26	.41*	–.04
	NI	–.35*	–.24	.13	–.37*	–.23	.13	–.16	.13	.07	–.37*	–.17	.14
Total	T	.24*	.36**	–.01	.32**	.36**	.08	.17	.12	–.10	.33**	.38**	–.01
	I	.27	.38	–.03	.42**	.44*	.21	.21	.21	–.03	.41**	.45*	.11
	NI	.13	.33*	.01	.19	.29	–.06	.03	.00	–.09	.17	.28	–.08

*Note*: ** *p* ≤ .001; * *p* ≤ .01. T = total sample, NI = non-Indigenous subgroup, I = Indigenous subgroup; Pre, post, and change ratings from the Violence Risk Scale-Sexual Offense version (VRS-SO) and Criminal Sentiments Scale (CSS) are correlated, respectively, with pre, post, and change ratings from the Structured Assessment of Protective Factors (SAPROF) measures. Pretreatment *n*s = 117–434 (T), 58–189 (I), 59–245 (NI); Posttreatment *n*s = 96–434 (T), 43–189 (I), 52–245 (NI); Change *n*s = 93–433 (T), 42–189 (I), 51–244 (NI). LCP = Law Courts Police, TLV = Tolerance for Law Violations, ICO = Identification with Criminal Others.

Finally, consistent with the risk principle, men in high intensity SOTP had significantly fewer protective factors (SAPROF total score) at pre (*M* = 10.3, *SD* = 4.9) and posttreatment (*M* = 14.4, *SD* = 5.5), than men in moderate intensity programs (pre *M* = 13.8, *SD* = 5.3; post *M* = 16.6, *SD* = 5.5), who, in turn, had fewer than those in low intensity programs (pre *M* = 18.1, *SD* = 5.4; post *M* = 19.3, *SD* = 5.4), *F* pre (2, 446) = 76.75, *p* < .001; *F* post (2, 446) = 25.48, *p* < .001. Further, larger SAPROF pre–post changes in protection were made by men in moderate (*M* = 2.8, *SD* = 3.2) and high (*M* = 4.2, *SD* = 4.2) intensity programs, than those men in low intensity programs (*M* = 1.2, *SD* = 4.2), *F* (2, 446) = 16.46, *p* <.001.

#### Associations With Responsivity Indicators of Prosocial and Adaptive Functioning

Table 5 reports convergent validity correlations between SAPROF total, subscale, and change scores, with several responsivity indicators including WAI-measured working alliance, indexes of cognitive ability, and vocational and educational attainment. SAPROF and WAI scores generally had weak (i.e., *r* < .10, or less than small) to small in magnitude (*r* = .10 to <.20), non-significant, positive correlations, indicating generally low convergence between the two measures. This was observed across SAPROF pretreatment, posttreatment, and change ratings with the WAI total and subscale scores for the aggregate sample and within ethnocultural subgroups. Although posttreatment ratings for SAPROF Motivational, External, and total scores had noticeably better convergence with WAI ratings than pretreatment scores, these correlations were not significant.

**Table 5.** Convergent Validity Correlations Between Convergent Responsivity Measure Scores With SAPROF Ratings as a Function of Indigenous Heritage.

Convergent responsivity measures		SAPROF measures											
	Group T/I/NI	Internal			Motivational			External			Total		
		Pre	Post	Change	Pre	Post	Change	Pre	Post	Change	Pre	Post	Change
Education	T	.32**	.31**	.04	.22**	.21**	–.01	.12	.19*	.05	.26**	.30**	.04
	I	.24*	.18	–.03	.18	.13	–.05	.11	.16	.03	.20	.19	–.03
	NI	.38**	.40**	.10	.20	.22	.03	.12	.23	.10	.28	.35**	.11
Employment	T	.24*	.27**	.07	.30**	.32**	.02	.25*	.22*	–.04	.35**	.35**	.02
	I	.21	.21	.05	.25	.21	–.04	.31*	.24	–.14	.33*	.27	–.06
	NI	.23	.27	.07	.31*	.40**	.10	.16	.25	.08	.32*	.41**	.11
WAI													
Total	T	.05	.07	.05	.13	.18	.05	.08	.13	.02	.13	.17	.06
	I	.02	.08	.10	.16	.14	–.01	–.03	–.11	–.13	.10	.07	–.02
	NI	.04	.04	.00	.09	.20	.11	.18	.33*	.13	.14	.26	.12
Task	T	.04	.09	.07	.12	.18	.08	.11	.15	.02	.12	.19	.09
	I	.04	.12	.14	.18	.18	.03	.05	–.05	–.14	.14	.13	.02
	NI	.01	.03	.02	.05	.17	.13	.15	.30*	.13	.09	.22	.15
Bond	T	.03	.07	.06	.13	.17	.03	.06	.11	.01	.13	.16	.04
	I	–.05	.03	.12	.12	.09	–.03	–.10	–.15	–.10	.02	.01	–.01
	NI	.08	.09	.01	.14	.24	.09	.21	.35**	.11	.09	.18	.09
Goal	T	.06	.06	.00	.11	.14	.03	.06	.12	.04	.11	.14	.04
	I	.10	.10	.03	.17	.13	–.03	–.01	–.08	–.14	.13	.08	–.06
	NI	.03	.00	–.04	.07	.14	.07	.13	.25	.11	.09	.18	.09
Cognitive Functioning													
CAAT RC	T	.15	.23	.16	.21	.28*	.09	.17	.19	.02	.23	.31*	.12
	I	.10	.08	.00	.25	.28	.05	.12	.22	.05	.22	.26	.06
	NI	.08	.26	.35	.07	.23	.22	.13	.27	.19	.11	.32	.33
Raven’s	T	.25*	.37**	.19	.35**	.33**	–.03	.13	–.02	–.11	.32**	.32**	–.02
	I	.39*	.31	.02	.45**	.31	–.12	.09	–.03	–.04	.39*	.28	–.11
	NI	.08	.33*	.35*	.26	.28	–.01	.13	–.09	–.21	.22	.24	–.01
SDMT	T	.24*	.20	–.04	.15	.22	.09	.09	.18	.08	.19	.27*	.09
	I	.28	.19	–.04	.23	.22	.02	.32	.17	–.17	.34*	.25	–.10
	NI	.23	.18	–.12	.13	.19	.06	–.05	.13	.17	.12	.23	.10
QT	T	.25*	.28*	.06	.34**	.31**	–.05	.16	.21	.05	.33**	.36**	.02
	I	.31	.17	–.09	.45**	.26	–.19	.08	–.04	–.02	.36*	.19	–.20
	NI	.14	.21	.07	.31	.26	–.11	.20	.27	.05	.29	.34*	–.01

*Note*: ** *p* ≤ .001; * *p* ≤ .01. T = total sample (*N* = 86–261), I = Indigenous subgroup (*n* = 45–138), NI = non-Indigenous subgroup (*n* = 41–121). WAI = Working Alliance Inventory, CAAT RC = Canadian Adult Achievement Test Reading Comprehension, SDMT = Symbol Digit Modality Test, QT = Quick Test.

By contrast, SAPROF scores showed generally better convergence with measures of cognitive functioning, indicating that higher levels of cognitive ability were associated with a greater number of protective factors (small to medium correlations). Specifically, SAPROF Internal, Motivational, and total scores (pre and post) were associated with greater levels of nonverbal reasoning (Raven’s scores) and verbal ability (Quick Test scores), with significant and broadly medium range correlations. Correlations with measures of reading achievement (CAAT) and processing speed (SDMT) were also in the small to medium range, and frequently, but not as consistently significant. By contrast, SAPROF External subscale scores were not significantly associated with any indicators of cognitive functioning, nor were correlations with any of the SAPROF change ratings. Finally, small to medium correlations were found between education and employment indicators with SAPROF pre and posttreatment scores across each subscale and the scale total; the only exception was a small and nonsignificant association between educational attainment and pretreatment External subscale ratings. Correlations were generally in the same category of effect size magnitude for Indigenous and non-Indigenous groups, but were frequently not significant owing to smaller cell sizes, and hence, power. Again, SAPROF change ratings had weak non-significant correlations with these specific responsivity indicators.

## Discussion

The present study examined the structural and convergent properties of SAPROF ratings in a large sample of treated Canadian men with sexual offense conviction histories. Our first objective was an examination of its factor structure and measurement invariance of factor loadings over two time points of administration. Our second main objective was to examine convergent associations through planned correlational analyses between scores on the SAPROF subscales and its total, with selected measures assessing conceptually relevant constructs across pre, post, and change ratings. These properties were examined within the aggregate sample and cross-validated across Indigenous and non-Indigenous groups.

### Factor Structure and Measurement Invariance

The results supported the measurement invariance and temporal stability of a modified three factor structure of SAPROF ratings over two measured time points. The results of a CFA of the original structure, and EFA to determine the best fitting structure, demonstrated the data to fit a different three-factor structure in the present sample compared to the original rationally derived three-subscale organization (Internal, External, Motivational) created by the developers (de Vogel et al., 2009). The results of ESEM affirmed a modified three-factor solution and the temporal stability of loadings, that was further demonstrated, via MG-ESEM, to be remarkably consistent across Indigenous and non-Indigenous groups. One reason for the discrepancies in SAPROF subscale structure from the original may reflect the use of different scale development procedures to form conceptual groupings; it is unsurprising that a rationally derived set of subscales (even those that make good sense) do not show an identical latent structure when examined factor analytically, especially one taken from a different offending sample, setting, context (treated), and country. In some respects, it may be remarkable that as many parallels were found between the solution that best fit these data, to the original three subscale model.

To the authors’ knowledge, this is the first study formally examining the factorial structure of SAPROF ratings across all items on the standalone tool (cf. Klepfisz et al., 2020). The latent structure of a tool, and the conceptual arrangement of its subscales, is not one and the same. For instance, the MMPI-2 has an extraordinarily complex latent structure underpinning its 567 items, with some solutions demonstrating as many as 21 separate factors (see Graham et al., 2022 for a review). However, a large varied set of psychometric procedures have gone into developing various sets of scales and subscales with considerable clinical utility, such as the empirical criterion keying approach used by Hathaway and McKinley (1942) to develop the original set of 10 basic clinical scales, or more recently, Tellegen et al.’s (2003) use of factor analytically grounded techniques to develop the psychometrically refined restructured clinical scales. In short, while the use of factor analytic techniques may refine scale content and organization, it need not constrain them.

The modified Internal-Prosocial factor in our sample corresponds to the strengths of a major risk-need domain counterpart from the Central Eight, antisocial personality pattern (Bonta & Andrews, 2024). The modified SAPROF factor embodies a collection of variables that include empathy, coping skills, self-control, motivation for treatment, positive attitude toward authority, and life goals which connote a significant protective effect for an individual’s ability to develop and maintain healthy social relationships, adherence to social norms, and engagement in prosocial activities while mitigating the risks associated with an antisocial personality pattern. In turn, the modified Motivational-Lifestyle factor reflects many general considerations that are indicative of prosocial lifestyle and intact prosocial problem-solving ability (e.g., intelligence, work, leisure activities), that reduce the likelihood of offending behaviors by mitigating lifestyle instability.

The External factor also underwent slight modification, thus retaining its name as professional care, living circumstances, and external control pertain to protective effects that are outside an individual’s control (de Vogel et al., 2011). Interestingly, the three items that loaded solely and highly on the External subscale (i.e., professional care, living circumstances, and external control) have been exclusively categorized together prior by the SAPROF’s developers (de Vries Robbé et al., 2011). For instance, de Vries Robbé et al. (2011) elucidated that elevated scores on items 15 through 17 denote a greater need for external control to manage an individual’s risk and is associated with a lower level of protective factors, whereas lower scores on the remaining items are associated with the opposite. Consequently, due to this prior categorization, one would anticipate that the original first two items of the External domain (i.e., social network and intimate relationship) and the latter three, would not necessarily fall under the same subscale. Thus, this predefined grouping by the developers aids in comprehending the observed divergence whereby only items 15 through 17 loaded exclusively and highly on the External subscale.

Finally, the results of an EFA of SAPROF change scores yielded an interesting pattern of findings. Changes in the most readily external items grouped together under a common construct, while changes in items traditionally grouped largely under the Internal and Motivational domains were captured under a common factor that reflected improvements in socioemotional and cognitive self-regulation and broader interpersonal and prosocial life functioning. Again, the results of ESEM within Indigenous and non-Indigenous groups showed reasonable continuity in this structure of SAPROF change scores. The clinical implications would seem to be that the interventions that stimulate growth in one set of psychologically based protective factors may have implications for the development in other areas. For instance, completing an attitudes and cognitions sexual offense group treatment module could foster the development of a more prosocial value system, ownership of one’s behavior in relationships (and greater understanding of the perspective and impacts on others), and the development of skills and strategies (e.g., challenging and correcting distorted thinking) that could improve cognitive self-control. Other treatment modules, vocational and educational opportunities, cultural healing rituals, and additional work therapeutically with allied professions could instill a common set of change processes across psychological domains.

### Convergent Associations With Measures of Risk and Need

Our second primary study aim was to examine the convergent validity of SAPROF factor and total scores with several conceptually relevant measures of risk and need across pre, post and change ratings. SAPROF measures correlated negatively with measures of sexual violence risk. These inverse correlations stand to reason as the scales were designed to capture constructs that are conceptually opposed; that is, individuals with a more serious sexual and nonsexual criminal history and who have a greater density and severity of criminogenic need, concordantly, have fewer protective factors. The associations with the factor domains of the VRS-SO helped contextualize the convergent properties. First, associations with Sexual Deviance tended to be smaller and less frequently significant, which is not unexpected given that this is a risk-need domain specific to sexual violence and which has no counterpart (e.g., strengths and resiliencies in sexual regulation) on the SAPROF, and was partly the impetus for development of the SAPROF-Sexual Offense Version (SAPROF-SO; Willis et al., 2017). The strong correlations with Criminality and Treatment Responsivity factor scores make sense conceptually, however. The VRS-SO Criminality factor is a generic risk-need factor that taps a broad propensity for crime and violence. It dovetails with many of the items across the SAPROF’s subscales, and most notably, the Motivational subscale. Moreover, the Treatment Responsivity factor essentially taps the attitudes and cognitions related to sexual offending and risk management, and accordingly, high scores on this domain would likely translate into deficits across the SAPROF domains. Such associations would likely yield some parallels to the SAPROF’s associations with a self-report measure of criminal attitudes.

SAPROF pre and post ratings for Internal, Motivational, and total scores also showed strong convergence with CSS subscales; specifically, greater protective factors were associated with the endorsement of fewer attitudes tolerant of law violations and identification with criminal associates (TLV and ICO subscales), and the endorsement of prosocial attitudes toward the law, court, and police (LCP subscale). By contrast, the weaker correlation between SAPROF External subscale and CSS measures may reflect the External subscale’s focus on environmental factors that are related to stability and support in the individual’s surroundings. The CSS, by contrast, primarily assesses internal cognitive factors such as attitudes, beliefs, and values, that may drive criminal behavior (Gendreau et al., 1979), with greater conceptual relevance to the SAPROF’s Internal and Motivation domains.

Finally, the frequent positive convergent associations between SAPROF change scores and dynamic change across the VRS-SO domains suggest at least some conceptual overlap between decreases in risk and increases in protective factors, measured from pre to posttreatment. In principle, targeting relevant dynamic risk factors in treatment should result in positive changes and potentially an increased presence of protective factors, as suggested by the meta-analytic findings of Burghart et al. (2023) where SAPROF change scores were associated with reduced violent and general recidivism. Thus, the positive correlations observed indicate the positive nature of therapeutic changes that are occurring and the SAPROF’s potential to have a role in monitoring treatment progress. The independence of changes in risk factors from changes in protective factors remains to be determined, however.

### The Responsivity Relevance of Protective Factor Ratings

A series of convergent validity analyses also examined the responsivity relevance of SAPROF ratings. First, SAPROF scores and self-report measures of the working alliance generally showed modest convergence, although this improved slightly for posttreatment ratings. The WAI primarily assesses the quality of the therapeutic relationship and the agreement between client and therapist on goals and tasks of therapy (Horvath & Greenberg, 1989). The SAPROF, on the other hand, is a measure of protective factors that represent individual characteristics or external supports that buffer risk for offending. While a strong therapeutic alliance could plausibly enhance the effectiveness of interventions aimed at promoting resilience, as demonstrated by the positive correlations, it is a distinct construct from protective factors. Further, a lack of protective factors should, in principle, have little bearing on the strength of the working alliance, as service providers ideally can develop sound alliances across clients with a range of strengths and resiliencies.

The SAPROF’s high convergence with measures of cognitive functioning and indicators of education and employment are consistent with the hypothesis and existing research indicating that higher cognitive ability serves as a protective effect against offending behavior (Nikulina & Widom, 2019; Ttofi et al., 2016). Further, given the content of the Internal and Motivational subscales, which encompass some protective factors associated with cognitive processing and motivation, stronger correlations may be anticipated than with external indicators of support. Of note, measures of verbal and nonverbal reasoning and educational attainment, which in and of themselves are protective and bode for treatment success, had more consistent associations with SAPROF scores, than measures of processing speed and reading achievement. Arguably, verbal and nonverbal reasoning may reflect broader psychosocial competence across personal and life domains that could translate into meeting one’s needs prosocially. Finally, employment history was positively correlated with SAPROF measures, especially the Motivational subscale which has work listed as a SAPROF domain. For instance, Porter et al. (2023) note that work history can act as a protective factor as employment limits the time available for an individual with criminal inclinations to pursue criminal activity when also socially embedded in an environment where they will adopt social values and engage in social learning.

### Ethnocultural and Practice Implications

Taken together, the factor structure, its temporal stability, and patterns of convergence with measures of risk, need, and responsivity are important psychometric properties of the SAPROF scores with practice implications in diverse correctional and forensic samples. First, the SAPROF factor structure is used to inform treatment planning by implementing a strength-based approach. The pattern of high and low scores across the three factors identifies an individual’s areas of strength and subsequently weaknesses, serving as an indication of where interventions should be targeted. Second, SAPROF change scores are essential in informing treatment progress, the changes occurring as treatment progresses should remain stable across the factors. As scores fluctuate, the factor structure should demonstrate stability. Establishing these properties bolsters confidence in clinical applications of the factor structure. Third, that levels of protection reliably correspond to levels of risk, the density and severity of criminogenic need, the amount of intervention related change, and other relevant and potentially salutary responsivity characteristics such as educational attainment, employment, and cognitive ability support the theoretical and clinical relevance of SAPROF ratings in correctional settings.

Fourth, cross-cultural support for the structural and convergent properties of SAPROF ratings affirms their potential utility with diverse correctional populations, particularly those that are overrepresented in correctional systems. Much of the controversy in forensic assessment with ethnocultural minorities is the adoption of a risk-centric lens that highlight problems associated with elevated risk, areas of deficit, and poor prospects for release and reintegration (Olver et al., 2024). The incorporation of protective factors into forensic assessment arguably prompts a mental shift in service providers to identify client strengths and resiliencies, treatment targets that are languaged in the direction of positive growth, and release and reintegration measures geared toward increasing the likelihood of positive outcomes (e.g., socioemotional wellbeing, employment stability, financial security, relationship harmony), in addition to preventing unwanted outcomes (i.e., recidivism).

### Strengths, Limitations, and Future Directions

There are notable strengths and limitations with implications for the generalizability of findings and future research. The present study featured a reasonably large sample of treated men with sexual offense histories, with generally complete and comprehensive ratings on the SAPROF and concordant measures, obtained at two timepoints. These methodological conditions were conducive to conducting an informative evaluation of the structural and convergent properties of SAPROF ratings, including the temporal stability of the factor structure in this sample. Moreover, this study to our knowledge, is the first to examine the factorial validity of the complete set of SAPROF item ratings as a standalone tool, which in turn, was done across two broad ethnocultural groups to extend support for these psychometric properties to Indigenous and non-Indigenous (White majority) correctional samples.

As a retrospective archival investigation, SAPROF ratings were dependent on the quality and the clarity of institutional files, which was generally strong, but nonetheless lacks a certain amount of clinical depth that likely contributed to some item omissions. In addition, the file content and organization and clinical data captured, were not typically done in a manner that emphasized protective factors; invariably, the files would be rather “risk centered” which likely placed some limits on the extraction of protective information. Further, although we had a reasonable sample for factor analysis, the availability of convergent validity variables, especially some responsivity measures, varied and likely contributed to some Type II errors; for instance, some correlations that were significant with certain measures (e.g., *r* = .15–.20s), were non-significant for others with smaller *n*. Finally, the results of factor analyses can vary depending on the extraction and rotation methods chosen as well as the sample, setting, and jurisdictional, and contextual factors. Moreover, given sample size constraints, we did not employ a split sample design to cross-validate the EFA and CFA on separate halves, nor did we have the resources to do an independent replication on separate samples. As an alternative, we utilized ESEM as an integration of EFA and CFA and utilized MG-CFA across ethnoracial groups as our approach to cross validation. While the solution we generated may be the best fitting model for a large, treated, Canadian, sexual offending sample in a correctional setting, it is unclear whether the same latent structure may be found in a forensic mental health sample of psychiatric inpatients in the Netherlands. These limitations taken together, replication on independent samples is essential and attempts at generalization are cautioned.

The findings warrant further research to evaluate the generalizability of the factor structure across diverse samples, beyond a Canadian sample of men with sexual offense conviction histories, and into different contexts and populations. Such work may elucidate nuances of the factor structure of SAPROF ratings, potentially bolstering its utility in clinical forensic risk assessments and across a range of contexts and demographics (Zilvinskis et al., 2017). Given that this is a single study, we would not yet advocate for adopting an alternate subscale structure to the tool in research or clinical practice. Such a direction would be one to be made by the instrument developers, and likely only after additional lines of factor analytic research would support a revision to the subscale structure of the tool. We do believe, however, that the External subscale, as it currently stands, seems to be fairly content heterogeneous, with the social network and intimate relationship items better captured by a motivational factor.

In closing, the factor structure, its temporal stability, and the convergent validity of SAPROF ratings are practical psychometric properties that are essential in providing support for the theoretical basis of the tool and the fundamental concepts it intends to measure. The findings broadly support the convergent validity of SAPROF ratings, indicating that the tool captures its targeted constructs, notwithstanding the results of factor analyses that demonstrated a less-than-perfect fit between the original and current item arrangement on its subscales and the latent structure emerging from our sample. This is not a criticism of the tool per se but rather may serve as an opportunity to enhance its efficacy and utility for future applications by way of factor analytic cross validations across different samples, settings, and jurisdictions.

## Data Availability

The study data cannot be shared publicly or with external researchers, given that the authors do not have IRB ethical approval or agency operational approval to do so. Researchers who may be interested in additional findings or data not presented in this manuscript may contact the corresponding author for additional analyses and any associated outputs. This study was not preregistered.
